# Hospital-acquired infections in preterm infants with gestational age <32 weeks: a retrospective study of clinical characteristics, pathogen distribution, and associated factors

**DOI:** 10.3389/fped.2026.1845022

**Published:** 2026-06-15

**Authors:** Yi-Mei Yang, Yan Dai, Yu-Yan Xie, Kun-Ling Song, Liu-Qing Li, Hui-He Tang, Di-Wen Zhang

**Affiliations:** Department of Pediatrics, The People’s Hospital of Guangxi Zhuang Autonomous Region, Nanning, China

**Keywords:** delayed cord clamping, infant, premature, nosocomial infection, peripherally inserted central catheter, risk factors, vitamin D deficiency

## Abstract

**Background:**

Preterm infants born at <32 weeks’ gestation are often highly susceptible to nosocomial infection owing to immune immaturity, prolonged hospitalisation, and frequent invasive procedures. Data on the epidemiology of infection and modifiable risk factors specific to this population in Chinese neonatal intensive care units (NICUs) remain limited.

**Methods:**

We conducted a retrospective cohort study of 170 preterm infants (<32 weeks’ gestation) admitted to the NICU of the People's Hospital of Guangxi Zhuang Autonomous Region between January 2022 and December 2024. The incidence of nosocomial infections, site distribution, and pathogen profiles were characterised. Risk and protective factors were evaluated using univariate and multivariate binary logistic regression. Model discrimination was assessed by receiver operating characteristic (ROC) curve analysis. The incidence of bronchopulmonary dysplasia (BPD), retinopathy of prematurity (ROP), preterm brain injury, and in-hospital mortality was compared between infected and non-infected infants.

**Results:**

Fifty-three infants (31.18%) developed nosocomial infections, yielding 61 infection episodes (episode rate 35.88%). Respiratory tract infections predominated (pneumonia 13.53%; ventilator-associated pneumonia 1.18%). Gram-negative bacteria accounted for 68.0% of isolates, with *Klebsiella pneumoniae* the most frequent species. In the multivariate model, peripherally inserted central catheter (PICC) catheterisation (adjusted OR = 3.172, 95% CI: 1.330–7.564) and 25-hydroxyvitamin D deficiency (adjusted OR = 2.867, 95% CI: 1.232–6.670) were independent risk factors, while delayed cord clamping (DCC) was independently protective (adjusted OR = 0.265, 95% CI: 0.113–0.619). The model AUC was 0.764 (95% CI: 0.693–0.836). BPD incidence was higher in the infection group than in the non-infection group (47.17% vs. 29.91%, *P* = 0.029); no between-group differences were observed for ROP, brain injury, or mortality.

**Conclusions:**

Our analysis shows that nosocomial infection affected approximately one-third of preterm infants <32 weeks’ gestation in this cohort. Three modifiable factors, i.e., PICC catheterisation, early vitamin D status, and DCC, were independently associated with infection risk. These findings support standardisation of PICC care bundles, early vitamin D supplementation, and routine DCC implementation as possible targets for infection prevention in this population.

## 1 Introduction

Advances in perinatal medicine and neonatal intensive care over the past two decades have markedly improved survival rates among preterm infants born before 32 weeks’ gestation (1). However, this group still faces a high risk of nosocomial infection. Several traits of extreme prematurity contribute to this vulnerability, such as the skin barrier being structurally and functionally immature, mucosal surfaces lacking a fully developed commensal microbiota, and both innate and adaptive immune systems being deficient in cell count, receptor diversity, and effector functions (2, 3). Prolonged NICU stays further increase these risks through repeated exposure to invasive devices, parenteral nutrition, broad-spectrum antibiotics, and frequent handling by multiple caregivers (4).

Reported nosocomial infection rates in very preterm and very low birth weight infants range from ca. 5.6%–34.4%, depending on the centre (hospital), surveillance definitions used, and population characteristics (5). The pathogens involved and their resistance profiles also differ across settings and over time. Several modifiable and non-modifiable risk factors have been identified in previous studies, including low birth weight, prolonged mechanical ventilation, central venous catheterisation, and duration of parenteral nutrition (6, 7). Recently, focus has shifted to perinatal interventions such as delayed umbilical cord clamping (DCC) and early nutritional supplementation, including vitamin D, as potentially protective exposures (8, 9), although evidence in infants <32 weeks remains limited and is largely derived from single-centre studies with varied designs.

In China, data on the epidemiology of nosocomial infections in preterm infants under 32 weeks’ gestation are limited. Existing reports reveal significant variation in infection rates and pathogen distributions, which reflect differences in NICU infrastructure, staffing ratios, clinical protocols, and diagnostic thresholds across institutions (10). It is uncertain whether risk factors identified in higher-resource settings apply similarly in Chinese NICUs. uncertain. There is limited evidence on the long-term impacts of nosocomial infections in this population, i.e., whether infections independently increase the risk of major morbidities like bronchopulmonary dysplasia (BPD), retinopathy of prematurity (ROP), or brain injury.

We therefore conducted a retrospective cohort study of preterm infants born at <32 weeks’ gestation and admitted to a single tertiary NICU over a three-year period (2022–2024). The aims are: (1) to describe the incidence, site distribution, and pathogen profile of nosocomial infections in this cohort; (2) to identify independent risk and protective factors using multivariable logistic regression; and (3) to assess the association between nosocomial infection and major neonatal complications, including BPD, ROP, preterm brain injury, and in-hospital mortality.

To our knowledge, our study is the first single-centre cohort in southern China to evaluate, in infants <32 weeks’ gestation, the joint contribution of PICC exposure, early postnatal 25(OH)D status, and DCC to nosocomial infection risk within a single multivariable framework, with consequent associations with BPD also examined.

## 2 Materials and methods

### 2.1 Study design and participants

This was a single-centre retrospective cohort study. We reviewed the medical records of all preterm infants with gestational age under 32 weeks and their mothers, admitted to the neonatal intensive care unit (NICU) at the People's Hospital of Guangxi Zhuang Autonomous Region between January 2022 and December 2024. A total of 177 infants were screened. Seven were excluded. Exclusions were due to: five because their hospital stay was less than 48 h; two because treatment was discontinued for non-medical (social) reasons. The final analysis included 170 infants (81 males, 89 females). Of these, 23 were born before 28 weeks of gestation, and 147 were born between 28 and 31⁺⁶ weeks. Birth weights ranged from 480 g to 1,870 g. Infants were categorised into an infection group and a non-infection group based on whether they developed a nosocomial infection during the index admission (see Section 2.4 for the case definition).

### 2.2 Eligibility criteria

**Inclusion criteria.** (1) Gestational age <32 weeks, confirmed by early ultrasound or last menstrual period; (2) duration of hospitalisation ≥48 h; (3) no evidence of infection present at the time of NICU admission.

**Exclusion criteria.** (1) Abandonment of treatment or death attributable to social factors rather than clinical deterioration; (2) major congenital malformations, confirmed genetic metabolic disorders, or chromosomal abnormalities; (3) missing data for key perinatal variables required for the analysis.

The study was approved by the Medical Ethics Committee of the People's Hospital of Guangxi Zhuang Autonomous Region (Approval No. KY-ZC-2024-227).

### 2.3 Data collection

We extracted all data retrospectively from the hospital electronic medical record system. Two categories of variables were collected.

**Infant variables:** sex, birth weight stratum (<1,000 g vs. ≥1,000 g), occurrence and duration of neonatal asphyxia, duration of invasive mechanical ventilation (categorised as none, ≤72 h, or >72 h), placement of an umbilical venous catheter (UVC), placement of a peripherally inserted central catheter (PICC), serum 25-hydroxyvitamin D [25(OH)D] concentration within the first postnatal week, red blood cell transfusion within the first postnatal week, complications during hospitalisation, and clinical status at discharge.

**Maternal perinatal variables:** Mode of delivery (vaginal vs. cesarean section), premature rupture of membranes, gestational diabetes mellitus, cervical insufficiency, Ureaplasma urealyticum infection, and whether delayed umbilical cord clamping (DCC) was performed at delivery.

### 2.4 Case definitions

**Nosocomial infection.** An infection that was neither present nor incubating at the time of NICU admission, diagnosed ≥48 h after admission or after the recognised average incubation period of the relevant pathogen, in accordance with the Ministry of Health diagnostic criteria (11).

**Late-onset sepsis (LOS).** Clinical diagnosis required abnormal clinical manifestations together with at least one of the following: (i) ≥2 abnormal non-specific blood markers; (ii) cerebrospinal fluid findings consistent with purulent meningitis; or (iii) detection of pathogenic bacterial DNA by high-throughput sequencing of blood. Definitive diagnosis required positive culture of blood or another normally sterile body fluid (e.g., cerebrospinal fluid) alongside compatible clinical features. Sepsis with onset at ≥3 days of age was classified as LOS (12).

**Pneumonia.** New pulmonary infection with onset ≥48 h after admission. Ventilator-associated pneumonia (VAP) was defined as a pulmonary infection occurring between 48 h after initiation of mechanical ventilation and 48 h after extubation (13).

**Necrotising enterocolitis (NEC).** Only cases meeting Modified Bell's staging criteria at stage II or above were included (14).

**Delayed umbilical cord clamping (DCC).** Clamping of the umbilical cord >60 s after delivery of the infant (15).

**Bronchopulmonary dysplasia (BPD) and retinopathy of prematurity (ROP).** Diagnosed according to the criteria in the 5th edition of Practical Neonatology (16).

**Preterm brain injury.** Defined as the presence of hydrocephalus, white matter injury, or grade III–IV periventricular–intraventricular haemorrhage on cranial ultrasound or magnetic resonance imaging.

**25(OH)D deficiency.** Serum 25(OH)D concentration <30 nmol/L, measured within the first postnatal week (17).

**Neonatal asphyxia.** A 1-minute or 5-minute Apgar score ≤7, together with umbilical cord blood pH <7.2 (18).

### 2.5 Microbiological methods

When nosocomial infection was clinically suspected, we collected specimens from the presumed site of infection (i.e., blood, cerebrospinal fluid, urine, respiratory secretions, or drainage fluid, as appropriate). Specimens were inoculated directly, or after enrichment, onto blood agar and/or selective agar plates and incubated under standard aerobic conditions for 48–72 h; cultures showing no growth were held for up to 6 days before being reported as negative. Species-level identification was performed using the BD Phoenix M50 automated microbial identification system (Becton Dickinson) for identification panels for Gram-negative (BD-N413) and Gram-positive (BD-P92) bacteria.

### 2.6 Statistical analysis

We performed all analyses in SPSS version 26.0 (IBM, USA). The significance level (*α*) was set at 0.05, and all tests were two-sided.

#### 2.6.1 Descriptive and univariate analyses

Continuous variables were assessed for normality using the Shapiro–Wilk test. Normally distributed data are presented as mean ± standard deviation (*x¯* ± *s*) and compared between groups using the independent-samples *t*-test. Non-normally distributed data are presented as median (interquartile range) [M (P25, P75)] and compared using the Mann–Whitney *U* test. Categorical variables are expressed as *n* (%) and compared using the Pearson *χ*^2^ test or, where expected cell frequencies were <5, Fisher's exact test.

Missing data: We applied complete-case analysis. Missingness for the key model variables [birth weight, neonatal asphyxia, DCC, PICC placement, 25(OH)D, and duration of mechanical ventilation] was <5% and apparently at random; bias from this approach is negligible.

Sample size: The final model contained 53 events and five predictors, yielding an events-per-predictor ratio of 10.3:1, commensurate with the conventional minimum of 10 for retrospective exploratory work.

#### 2.6.2 Univariate logistic regression

Variables with *P* < 0.10 in univariate analyses, supplemented by variables selected on clinical grounds, were entered individually into univariate binary logistic regression models, with nosocomial infection (present = 1, absent = 0) as the dependent variable. Crude odds ratios (ORs) and their 95% confidence intervals (CIs) were calculated.

#### 2.6.3 Multivariate logistic regression

Variables retaining statistical significance in the univariate logistic regression, together with any variables judged clinically essential *a priori*, were entered simultaneously (forced-entry method) into a multivariate binary logistic regression model. Multicollinearity was assessed in SPSS 26.0 using the variance inflation factor (VIF); values ≥10 were considered indicative of problematic collinearity. Overall model validity was assessed by the omnibus likelihood-ratio test. Model calibration was evaluated using the Hosmer–Lemeshow goodness-of-fit test; *P* > 0.05 was considered an acceptable fit. Adjusted ORs and 95% CIs were reported for each covariate.

#### 2.6.4 Predictive model evaluation

A receiver operating characteristic (ROC) curve was constructed from the multivariate model's predicted probabilities. The area under the curve (AUC) and its 95% CI were calculated. The optimal probability threshold was determined by the maximum Youden index (*J* = sensitivity + specificity − 1), and the corresponding sensitivity, specificity, positive predictive value, and negative predictive value were reported.

#### 2.6.5 Outcome comparisons

The incidence of BPD, ROP, preterm brain injury, and in-hospital mortality was compared between the infection and non-infection groups using the *χ*^2^ test or Fisher's exact test, as appropriate.

## 3 Results

### 3.1 Nosocomial infection incidence and pathogen distribution

Of the 170 preterm infants with gestational age <32 weeks, 53 (31.18%) developed nosocomial infections, yielding 61 infection episodes (including infants with dual infections) and an episode rate of 35.88% (61/170). The total hospital days were 8,154, corresponding to a daily nosocomial infection incidence of 0.75% (61/8,154). Eight of the 53 infected infants experienced two separate infection episodes.

Respiratory tract infections predominated. Pneumonia occurred in 23 infants (13.53%), accounting for 25 episodes, of which two were ventilator-associated pneumonia (VAP). Late-onset sepsis (LOS) was diagnosed in 18 infants (10.59%); 4 of these 18 (22.22%) had concurrent purulent meningitis. Necrotising enterocolitis (NEC) occurred in 11 infants (6.47%), with 2 of 11 (18.18%) complicated by purulent meningitis. One infant (0.59%) developed a urinary tract infection (Supplementary Table S1).

The bacterial culture submission rate was 88.52% (54/61 episodes), and the positivity rate among submitted cultures was 46.30% (25/54). Twenty-five pathogenic strains were isolated; no fungi were detected. Gram-negative bacteria accounted for 17 strains (68.0%), with *K. pneumoniae* being the most frequently isolated species (7 strains; 41.18% of Gram-negative isolates). Gram-positive bacteria accounted for the remaining 8 strains (32.0%; Supplementary Table S2).

### 3.2 Univariate analysis

Chi-squared tests identified five variables with differential distribution between the infection and non-infection groups (*P* < 0.05): birth weight (<1,000 g vs. ≥ 1,000 g), neonatal asphyxia, delayed cord clamping (DCC), PICC catheterisation, and 25-hydroxyvitamin D [25(OH)D] deficiency. Gender, mode of delivery, premature rupture of membranes, gestational diabetes, cervical insufficiency, Ureaplasma urealyticum infection, duration of mechanical ventilation, umbilical venous catheter (UVC) catheterisation, and red blood cell transfusion within the first week did not differ between groups (all *P* > 0.05; Table 1).

**Table 1:** Univariate analysis of factors affecting nosocomial infections in preterm infants with gestational age <32 weeks.

Group	Infection Group	Non-infection Group	*χ* ^2^	*P*
Number of cases	53	117		
Gender (cases)			3.630	0.057
Male	31	50		
Female	22	67		
Birth weight [*n* (%)]			4.658	0.031
<1,000 g	18 (33.96)	22 (18.80)		
≥1,000 g	35 (66.04)	95 (81.20)		
Premature rupture of membranes [*n* (%)]	16 (30.19)	40 (34.19)	0.264	0.607
Gestational diabetes [*n* (%)]	13 (24.53)	35 (29.91)	0.522	0.470
Cervical insufficiency [*n* (%)]	20 (37.74)	22 (18.80)	0.241	0.624
Mode of delivery [*n* (%)]			0.186	0.666
Vaginal delivery	19 (35.85)	38 (32.48)		
Cesarean section	34 (64.15)	79 (67.52)		
Neonatal asphyxia [*n* (%)]	20 (37.74)	22 (18.80)	7.029	0.008
Duration of mechanical ventilation [*n* (%)]			0.976	0.614
No	30 (56.60)	73 (62.39)		
≤72 h	7 (13.21)	17 (14.53)		
>72 h	16 (30.19)	27 (23.08)		
Delayed umbilical cord clamping [*n* (%)]	10 (18.87)	59 (50.43)	15.066	<0.001
UVC catheterization [*n* (%)]	41 (77.36)	81 (69.23)	1.189	0.275
PICC catheterization [*n* (%)]	44 (83.02)	68 (58.12)	10.061	0.002
25 (OH)D deficiency [*n* (%)]	18 (33.96)	18 (15.38)	7.542	0.006
Red blood cell transfusion within 1 week [*n* (%)]	17 (32.08)	33(28.21)	0.263	0.608

### 3.3 Univariate logistic regression

Variables reaching *P* < 0.10 in the chi-squared analysis were entered into univariate logistic regression models with nosocomial infection as the dependent variable (1 = infection, 0 = no infection) (Supplementary Table S3. Birth weight <1,000 g (OR = 2.221, 95% CI: 1.066–4.625), neonatal asphyxia (OR = 2.617, 95% CI: 1.269–5.396), PICC catheterisation (OR = 3.523, 95% CI: 1.574–7.884), and 25(OH)D deficiency (OR = 2.829, 95% CI: 1.325–6.039) were each associated with increased odds of infection, whereas DCC (OR = 0.229, 95% CI: 0.105–0.498) was associated with reduced odds (Table 2).

**Table 2:** Univariate logistic regression analysis of factors affecting nosocomial infections in preterm infants with gestational age <32 weeks.

Variable	*β*	*SE*	Wald *χ*^2^	*P*	*OR*	OR (95%CI)	
						Lower	Upper
Birth weight	0.798	0.374	4.543	0.033	2.221	1.066	4.625
Neonatal asphyxia	0.962	0.369	6.791	0.009	2.617	1.269	5.396
Delayed cord clamping (DCC)	−1.476	0.397	13.831	<0.001	0.229	0.105	0.498
PICC catheterisation	1.259	0.411	9.386	0.002	3.523	1.574	7.884
25 (OH)D deficiency	1.040	0.387	7.218	0.007	2.829	1.325	6.039

### 3.4 Multivariate logistic regression

All five variables from the univariate regression were entered into a multivariate binary logistic regression model using the forced-entry method. The overall model was statistically significant (omnibus test *χ*^2^ = 33.957, df = 5, *P* < 0.001) and demonstrated adequate fit (Hosmer–Lemeshow test *χ*^2^ = 7.761, df = 8, *P* = 0.457). Variance inflation factors ranged from 1.0 to 1.1 across all covariates, indicating no substantial multicollinearity. VIF values were 1.150 (birth weight), 1.140 (DCC), 1.111 (neonatal asphyxia), 1.063 (PICC catheterisation), and 1.025 [25(OH)D deficiency]; none approached the conventional threshold of 10.

PICC catheterisation (adjusted OR = 3.172, 95% CI: 1.330–7.564, *P* = 0.009) and 25(OH)D deficiency (adjusted OR = 2.867, 95% CI: 1.232–6.670, *P* = 0.014) remained independently associated with nosocomial infection. DCC (adjusted OR = 0.265, 95% CI: 0.113–0.619, *P* = 0.002) remained independently protective. Birth weight and neonatal asphyxia were no longer statistically significant in the adjusted model (both *P* > 0.05; Table 3).

**Table 3:** Multivariate logistic regression analysis of factors affecting nosocomial infections in preterm infants with gestational Age <32 weeks.

Variable	*β*	*SE*	*Wald χ* ^2^	*P*	*OR*	OR (95%CI)	
						Lower	Upper
Birth weight	0.035	0.429	0.006	0.936	1.035	0.446	2.401
Delayed cord clamping (DCC)	−1.328	0.433	9.409	0.002	0.265	0.113	0.619
Neonatal asphyxia	0.662	0.413	2.572	0.109	1.939	0.863	4.355
PICC atheterization	1.154	0.443	6.778	0.009	3.172	1.330	7.564
25 (OH)D deficiency	1.053	0.431	5.977	0.014	2.867	1.232	6.670

### 3.5 Predictive model performance

**Figure 1:**
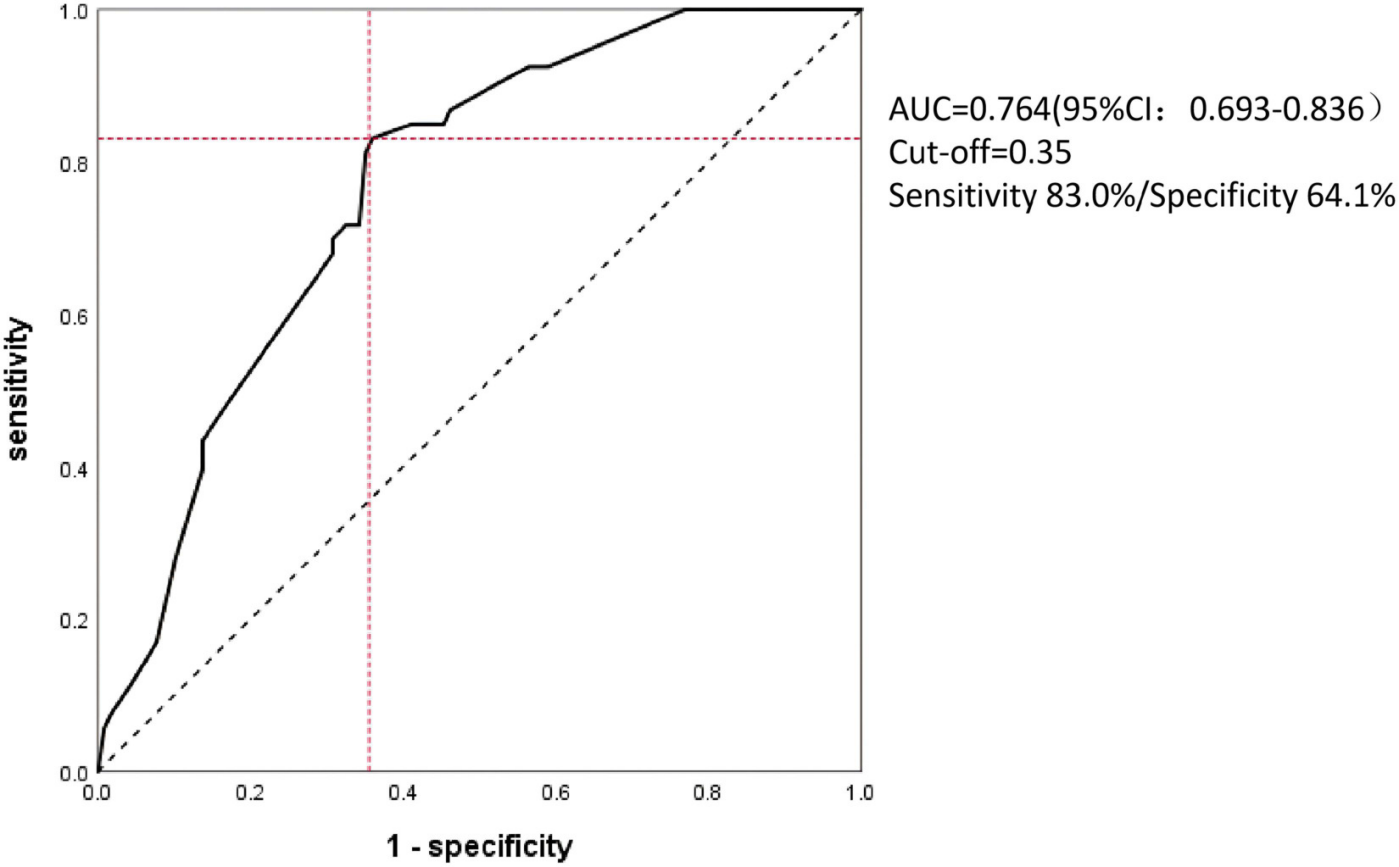
ROC curve of the multi-factor logistic regression model forhospital infection in premature infants with gestational age <32 weeks.

The receiver operating characteristic (ROC) curve for the multivariate model yielded an area under the curve (AUC) of 0.764 (95% CI: 0.693–0.836, *P* < 0.001). The optimal probability threshold, determined by maximum Youden index (0.471), was 0.35, at which point sensitivity was 83.0% and specificity 64.1% (Figure 1).

### 3.6 Association between nosocomial infection and clinical outcomes

The incidence of bronchopulmonary dysplasia (BPD) was higher in the infection group than in the non-infection group (47.17% vs. 29.91%, *χ*^2^ = 4.756, *P* = 0.029). No statistically significant between-group differences were observed for retinopathy of prematurity (ROP; 7.55% vs. 7.69%, *P* = 0.974), preterm brain injury (13.21% vs. 11.97%, *P* = 0.820), or mortality (11.32% vs. 7.69%, *P* = 0.631; Table 4). We report in-hospital mortality as all-cause, not infection-attributable.

**Table 4:** Complications and outcomes in preterm infants with gestational age <32 weeks [*n* (%)].

Complication/Outcome	Infection group	Non-infection group	*χ* ^2^	*P*
ROP	4 (7.55)	9 (7.69)	0.001	0.974
BPD	25 (47.17)	35 (29.91)	4.756	0.029
Preterm brain injury	7 (13.21)	14 (11.97)	0.052	0.820
Mortality	6 (11.32)	9 (7.69)	0.231	0.631

## 4 Discussion

In this retrospective study of 170 preterm infants born at <32 weeks’ gestation, the nosocomial infection rate was 31.18%, with an episode rate of 35.88% and a daily incidence of 0.75%. Multivariate logistic regression identified PICC catheterisation and 25(OH)D deficiency as independent risk factors, while DCC was independently protective. The predictive model showed moderate discriminative ability (AUC = 0.764). Among complications, only BPD was more common in the infection group. These findings are discussed in the context of existing evidence, with a focus on biological plausibility and study limitations.

### 4.1 Infection rates and pathogen distribution

The observed infection rate of 31.18% falls within the 5.6–36.6% range reported across centres with different resource levels and geographic locations (5, 19–21). The wide variation in published rates likely reflects differences in diagnostic thresholds, surveillance intensity, and baseline population risk profiles rather than any single factor. Respiratory tract infections were the most common, consistent with impaired mucociliary clearance, reduced airway secretion mobilisation, and frequent exposure to invasive respiratory support, all of which are characteristic of infants at this gestational age (22, 23).The high proportion of LOS cases (22.22%) and NEC cases (18.18%) with concurrent purulent meningitis warrants emphasis. The blood–brain barrier in preterm infants at <32 weeks’ gestation is structurally immature and exhibits increased paracellular permeability, which facilitates haematogenous seeding of the central nervous system during bacteraemia. This finding supports the routine inclusion of cerebrospinal fluid examination in the diagnostic workup of infants presenting with LOS or NEC at this gestational age.

Gram-negative organisms accounted for 68.0% of isolates, with *K. pneumoniae* the most common species, at 41.18% of Gram-negative strains. This distribution is consistent with national neonatal surveillance data (24, 25). *K. pneumoniae* colonizes the gastrointestinal tract and is easily transmitted through healthcare workers’ hands and contaminated equipment surfaces. Its ability to form biofilms on indwelling devices further complicates eradication (26). The dominance of Gram-negative pathogens has direct implications for the choice of empirical antibiotics and underscores the importance of hand hygiene, surface disinfection, and barrier precautions in preventing horizontal transmission.

### 4.2 Delayed cord clamping as a protective factor

DCC was independently associated with reduced odds of nosocomial infection (adjusted OR = 0.265). This finding is consistent with data from randomised controlled trials and meta-analyses showing reduced rates of both NEC and LOS following DCC (27, 28). The mechanism is reasonable for several reasons. Delaying cord clamping by more than 60 s allows continued placental–fetal blood transfer, increasing circulating blood volume, hematocrit, and iron stores at birth (29). The elevated blood volume boosts circulating neutrophil and lymphocyte counts, enhancing innate immune function during the early postnatal period (30). DCC also enhances the transfer of hematopoietic stem cells and cytokines from placental blood, which may support organ maturation after birth and reduce susceptibility to infections (31).

In this cohort, the DCC implementation rate was 18.87% in the infection group, compared with 50.43% in the non-infection group. This disparity likely reflects, at least in part, confounding by indication, that is, infants delivered under emergency conditions (e.g., placental abruption, severe fetal bradycardia) are less likely to receive DCC and are independently at higher baseline risk for infection. The magnitude of the protective effect observed here (OR = 0.265) may therefore overestimate the true causal contribution of DCC. Adjustments for delivery urgency and immediate postnatal condition would be required to more precisely isolate the independent effect of DCC. Nonetheless, the direction of effect is biologically coherent and consistent with trial-level evidence. Knol et al. demonstrated that physiologically based cord clamping can be performed using a purpose-built resuscitation trolley, permitting concurrent stabilisation without immediate cord severance (32). Broader adoption of such equipment may increase DCC rates in preterm deliveries, where it is currently omitted due to perceived constraints on resuscitation.

### 4.3 PICC catheterisation as a risk factor

PICC catheterisation was the independent risk factor in the multivariate model (adjusted OR = 3.172), consistent with previous reports (33, 34). The underlying mechanisms are well understood. Percutaneous catheter insertion damages the skin barrier, creating a direct entry point for skin and environmental microbes. Once in place, the catheter surface serves as a substrate for biofilm formation, i.e., structured microbial communities embedded in an extracellular polymeric matrix that confers resistance to both immune clearance and antimicrobial agents (35). In infants under 32 weeks’ gestation, the small size of peripheral and central vessels makes insertion more technically challenging and increases the risk of endothelial injury, which can lead to local inflammation and bacterial adhesion (36). Rapid growth in this group also heightens the risk of catheter tip migration over time, potentially leading to malpositioning (37). Longer dwell times and repeated hub manipulation further raise these risks by increasing exposure to microbial colonisation and the intraluminal migration of organisms (38).

Infants who received PICC placement in our cohort tended to have lower gestational age, lower birth weight, greater illness severity, and poorer peripheral venous access; indication bias is therefore likely. We modelled PICC as a dichotomous exposure, and the adjusted set comprised birth weight (≥1,000 g vs. <1,000 g), neonatal asphyxia, and DCC. Gestational age as a continuous variable, ventilation duration, and a validated illness severity score were not entered. Ipso facto, we cannot fully disentangle the catheter effect from underlying severity, and the observed association probably reflects, in part, residual confounding. Prospective work should treat PICC dwell time as continuous and incorporate a validated severity score adduced ex ante to catheter insertion.

From a clinical perspective, reducing the risk of catheter-associated infections relies on several modifiable practices: strict adherence to maximum sterile barrier precautions during insertion, use of ultrasound guidance to minimise insertion attempts, limiting the number of operators involved, achieving full enteral feeding early (especially with breast milk) to shorten catheter dwell time (39), and promptly removing the catheter at the first signs of infection.

### 4.4 25-hydroxyvitamin D deficiency as a risk factor

Serum 25(OH)D deficiency within the first postnatal week was independently associated with nosocomial infection (adjusted OR = 2.867). Vitamin D exerts immunomodulatory effects primarily through the vitamin D receptor (VDR), which is expressed on most immune cell lineages. Ligand binding at the VDR upregulates expression of antimicrobial peptides, notably cathelicidin (LL-37) and defensins, on mucosal and epithelial surfaces. These peptides exhibit broad-spectrum antimicrobial activity against Gram-negative and Gram-positive bacteria, including drug-resistant strains (40, 41).

Vitamin D also modulates macrophage and dendritic cell function, enhancing phagocytic capacity and antigen presentation while attenuating excessive pro-inflammatory cytokine release (42). In preterm infants, whose adaptive immune systems are functionally immature, these innate immune mechanisms constitute a particularly critical component of host defence. Deficiency in 25(OH)D therefore reduces the functional capacity of this innate barrier, increasing vulnerability to nosocomial pathogens.

### 4.5 Association between nosocomial infection and BPD

BPD incidence was higher in the infection group (47.17%) than in the non-infection group (29.91%; *P* = 0.029). This observation is consistent with evidence linking postnatal sepsis to pulmonary inflammation and subsequent dysregulated alveolar and vascular development characteristic of BPD (43). The putative mechanism involves the systemic and pulmonary release of pro-inflammatory cytokines (e.g., IL-1β, IL-6, TNF-α) during infection, which disrupt the tightly regulated signalling cascades that govern late-stage alveolarisation and pulmonary microvasculature maturation (44).

This association should be interpreted with caution. The analysis was unadjusted, and the infection–BPD relationship is plausibly bidirectional: prolonged mechanical ventilation and supplemental oxygen exposure militate towards both outcomes. The comparison spanned four secondary endpoints without correction for multiple testing; ceteris paribus, the *P*-value of 0.029 would not survive a Bonferroni-corrected threshold of 0.0125. A multivariable analysis adjusting for gestational age, birth weight, and cumulative ventilatory exposure would be required to assess whether nosocomial infection independently raises BPD risk in this cohort.

No between-group differences were detected for ROP, preterm brain injury, or mortality, though the sample size may have been insufficient to detect clinically meaningful differences in these lower-frequency outcomes.

We note several limitations of our study. First, the single-centre retrospective design limits generalisability. Centre-specific practices, pathogen ecology, and patient demographics may differ from those in other NICUs. Second, several established risk factors were not included in the model, notably parenteral nutrition duration, antibiotic exposure, feeding type, and gestational age as a continuous variable. Residual confounding from these unmeasured or incompletely modelled variables cannot be excluded. Finally, model discrimination, while acceptable (AUC = 0.764), is moderate, and the model's clinical utility for individual risk stratification remains to be demonstrated prospectively.

### 4.6 Conclusions

Nosocomial infections affected about one third of preterm infants <32 weeks’ gestational age in this single-center study, with respiratory tract infections and Gram-negative organisms, particularly K. pneumoniae, being most common. PICC catheterisation and early postnatal 25(OH)D deficiency were independently associated with an increased risk, while DCC was protective. These three factors can be modified at the bedside. Standardising PICC insertion and care, starting early vitamin D supplements, and routinely applying DCC in eligible preterm deliveries are practical interventions. The link between nosocomial infection and BPD, although needing confirmation in adjusted analyses, highlights the broader clinical impact of infection in this group. Larger multicenter prospective studies with validated severity scores and predefined adjustments for confounders are needed to validate these findings and improve the predictive model.

## Data Availability

The raw data supporting the conclusions of this article will be made available by the authors, without undue reservation.
