# Supplementary material for: Hospital-acquired infections in preterm infants with gestational age <32 weeks: a retrospective study of clinical characteristics, pathogen distribution, and associated factors
Source: Front Pediatr. 2026 Jun 15;14:1845022. doi: 10.3389/fped.2026.1845022 (PMC13310890; doi:10.3389/fped.2026.1845022)
Supplement: Supplementary file 1 [file Table1.docx]

**Supplementary Information File**

**Hospital-Acquired Infections in Preterm Infants With Gestational Age <32 Weeks: A Retrospective Study of Clinical Characteristics, Pathogen Distribution, and Associated Factors**

Yi-Mei Yang^1,†^; Yan Dai^1,†^, Yu-yan Xie^1^, Kun-ling Song^1^, Liu-Qing Li^1^,Di-Wen Zhang^1,*^

^1^ Department of Pediatrics, the People’s Hospital of Guangxi Zhuang Autonomous Region, Nanning, China

^†^ These authors contributed equally to this work and share first authorship

^*^ Corresponding author E-mail: [Dr.Zdiwen@outlook.com](mailto:Dr.Zdiwen@outlook.com)

_____

| Table 1 Distribution of Hospital-Acquired Infection Sites in Preterm Infants with Gestational Age <32 Weeks (Including Cases with Dual Infections)‌ | | |
| --- | --- | --- |
| Hospital-Acquired Infection Site | Number of Episodes (n) | Percentage（%） |
| Pneumonia  VAP | 23  2 | 37.70  3.28 |
| LOC | 18 | 29.51 |
| NEC | 11 | 18.03 |
| Pyogenic Meningitis | 6 | 9.84 |
| Urinary Tract Infection | 1 | 1.59 |

| Table 2 Characteristics of Pathogen Distribution in Hospital-Acquired Infections among Preterm Infants with Gestational Age <32 Weeks | | |
| --- | --- | --- |
| Pathogen | Number of Strains (n) | Percentage（%） |
| ‌Gram-Positive Bacteria |  |  |
| Staphylococcus aureus | 1 | 4.00 |
| Staphylococcus epidermidis | 1 | 4.00 |
| Staphylococcus haemolyticus | 3 | 12.00 |
| Streptococcus agalactiae | 1 | 4.00 |
| Enterococcus faecium（E. faecium） | 2 | 8.00 |
| Gram-Negative Bacteria |  |  |
| Escherichia coli | 3 | 12.00 |
| Klebsiella pneumoniae | 7 | 28.00 |
| Pseudomonas aeruginosa | 4 | 16.00 |
| Serratia marcescens | 2 | 8.00 |
| Klebsiella oxytoca | 1 | 4.00 |

| Table 3 Assignment of Variables for Factors Associated with Hospital-Acquired Infections in Preterm Infants with Gestational Age <32 Weeks | |
| --- | --- |
| Factor | Assignment |
| Birth weight | ＜1000=0,≥1000=1 |
| Neonatal asphyxia | Absent = 0, Present = 1 |
| Delayed cord clamping | No = 0, Yes = 1 |
| PICC placement | Absent = 0, Present = 1 |
| 25(OH)D deficiency | Absent = 0, Present = 1 |
